# Urgent endovascular aortic dissection repair in a Marfan patient during COVID‐19 pandemic

**DOI:** 10.1002/ccr3.4634

**Published:** 2021-08-15

**Authors:** Xun Yuan, Mireya Castro Verdes, Massimo Capoccia, Ulrich Rosendahl, Christoph A. Nienaber

**Affiliations:** ^1^ Cardiology and Aortic Centre Royal Brompton & Harefield NHS Foundation Trust London England; ^2^ National Heart and Lung Institute Imperial College London London England; ^3^ Imaging Department Royal Brompton & Harefield NHS Foundation Trust London England; ^4^ Cardiac Surgery and Aortic Centre Royal Brompton & Harefield NHS Foundation Trust London England

**Keywords:** aortic dissection, COVID‐19, Marfan's syndrome, thoracic endovascular repair

## Abstract

Although Thoracic Endovascular Aortic Repair is usually applied to patients without connective tissue disorders, our case shows its potential for complicated type B aortic dissection in a Marfan patient as a feasible alternative to open redo surgery with good short‐term outcomes.

## Graphical abstract

Endovascular management using modern highly flexible endograft technology and advanced image guidance may be useful for complex aortic dissection in Marfan patients in post‐pandemic times.

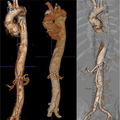

## 1 INTRODUCTION

We present a successful Thoracic Endovascular Aortic Repair (TEVAR) procedure for acute complex type B aortic dissection in a Marfan patient during COVID‐19 pandemic while cardiac surgery was not readily available. TEVAR procedure was performed in lieu of open redo surgery to avoid long intensive care.

Marfan's syndrome (MFS) is a heritable connective tissue disorder affecting skeletal, ocular, and cardiovascular systems. Patients with MFS are prone to develop vascular complications such as aortic dilatation, dissection, and rupture.^1^, ^2^ The COVID‐19 pandemic during the first wave caused a major disruption of elective and even urgent surgical services in London. A combination of severely restricted resources, during this period and a reluctance of patients to seek medical assistance in fear of COVID‐19, have led to delayed or missed affection to even life‐threatening conditions, including acute aortic syndromes.^3^

## 2 CASE DESCRIPTION

**FIGURE 1:**
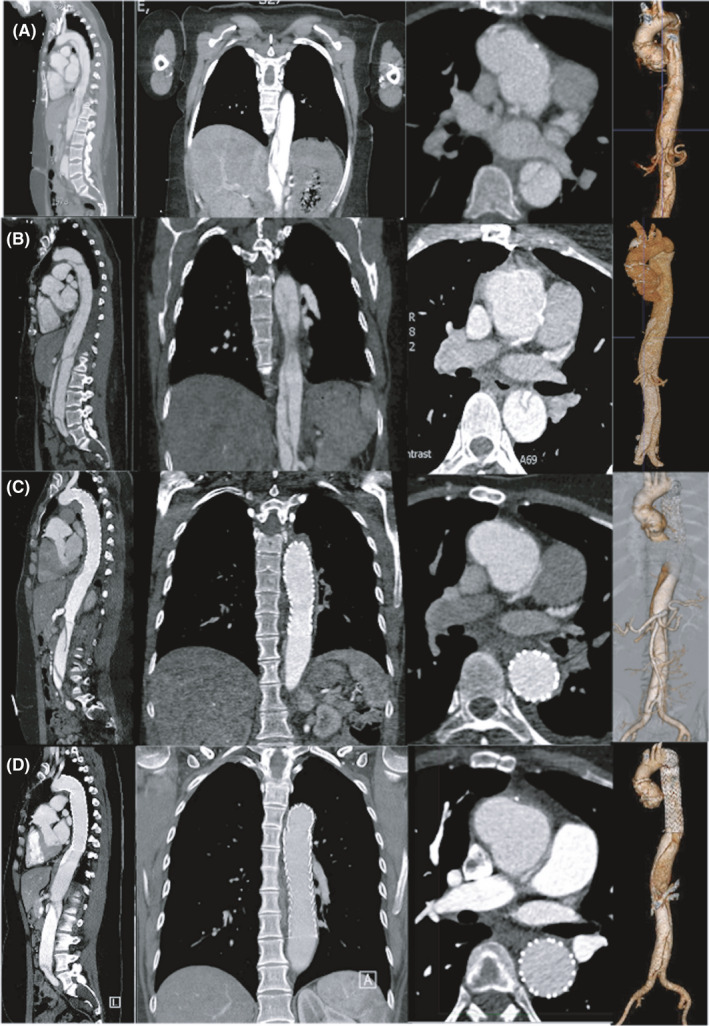
Diagnostic contrast computed tomography (CT) shows acute type B aortic dissection with a flap originating at the origin of the left subclavian artery and extending beyond the aortic bifurcation into both common iliac arteries (panel A); follow‐up CT at day 7 reveals further dilatation of the dissected descending thoracic aorta with compression of the true lumen at mid‐descending thoracic aorta level (panel B); Post‐TEVAR CT demonstrates complete exclusion of the false lumen without malperfusion (panel C); follow‐up CT at 7 months shows an excellent result and aortic remodelling with no distal expansion of the abdominal remainder of the dissection (panel D)

A 33‐year‐old patient presented with sudden severe central chest pain radiating to the back on May 31, 2020. She had been suspected with the diagnosis of Marfan syndrome on clinical grounds in 2009 and genetically confirmed in 2011 with a FBN1 gene mutation; she represents the first spontaneous mutation in the family with no other family members affected. With confirmed diagnosis, she underwent elective aortic root replacement in 2013 for root aneurysm and received a 29 mm Freestyle™ Stentless bioprosthesis (Medtronic Inc.), left coronary reconstruction and replacement of the ascending aorta using a 32 mm Gelweave™ Dacron graft (Terumo Vascutek Ltd.). Ever since she remained under annual surveillance in the aortopathy clinic. With sudden onset of severe chest pain on May 31, 2020, contrast enhanced computed tomography (CT) revealed acute type B aortic dissection with a flap originating 16 mm beyond the origin of the left subclavian artery and extending beyond the aortic bifurcation into both common iliac arteries (Figure 1A). The flap presented in a spiral fashion with the celiac trunk, superior mesenteric and left renal artery originating from the false lumen, accompanied with abdominal pain and weak pulses in both legs.

Under conditions of the raging COVID‐19 pandemic immediate transfer to an aortic center for surgical treatment was not available for some time and optimized medical management was commenced in intensive care with permissive hypotension by intravenous labetalol infusion and oral Losartan despite clinical signs of malperfusion. Repeat CT imaging was prompted in view of recurrent back pain and abdominal discomfort and revealed further dilatation of the dissected descending thoracic aorta with compression of the true lumen at mid‐descending thoracic aorta level (Figure 1B). With these evolving features of complicated dissection and two emergency sessions of multidisciplinary hub meetings involving tertiary surgical centers in London, an endovascular approach was considered appropriate and technically feasible to minimize the risk of both a prolonged hospital stay after open surgery, and potential exposure to SARS‐Cov‐2 virus.

## 3 INTERVENTION

**FIGURE 2:**
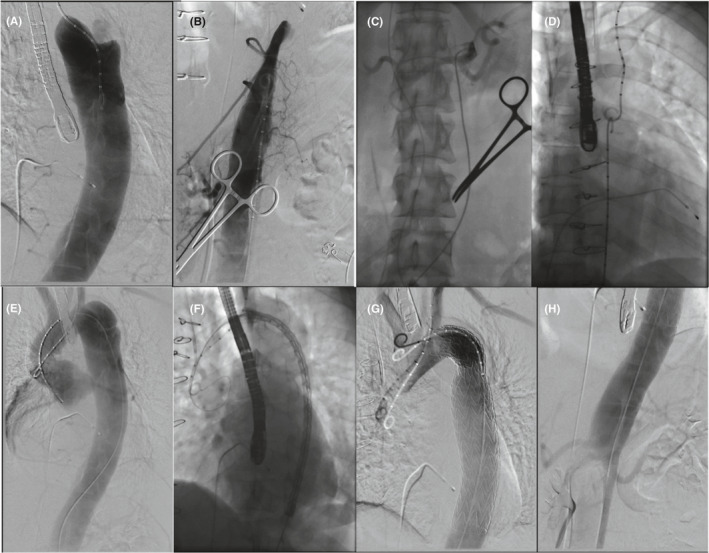
Marker pigtail catheter from left brachial artery access to identify the proximal true lumen (A) pigtail catheter from right femoral artery access (B) confirmation of true lumen by selective imaging of celiac trunk (C) “embracing pigtails” to confirm a true lumen connection (D) aortogram to re‐assess dissected segment via pigtail (E) stent graft positioning under fluoroscopy and TOE (F) aortogram to review results after deployment (G, H)

**FIGURE 3:**
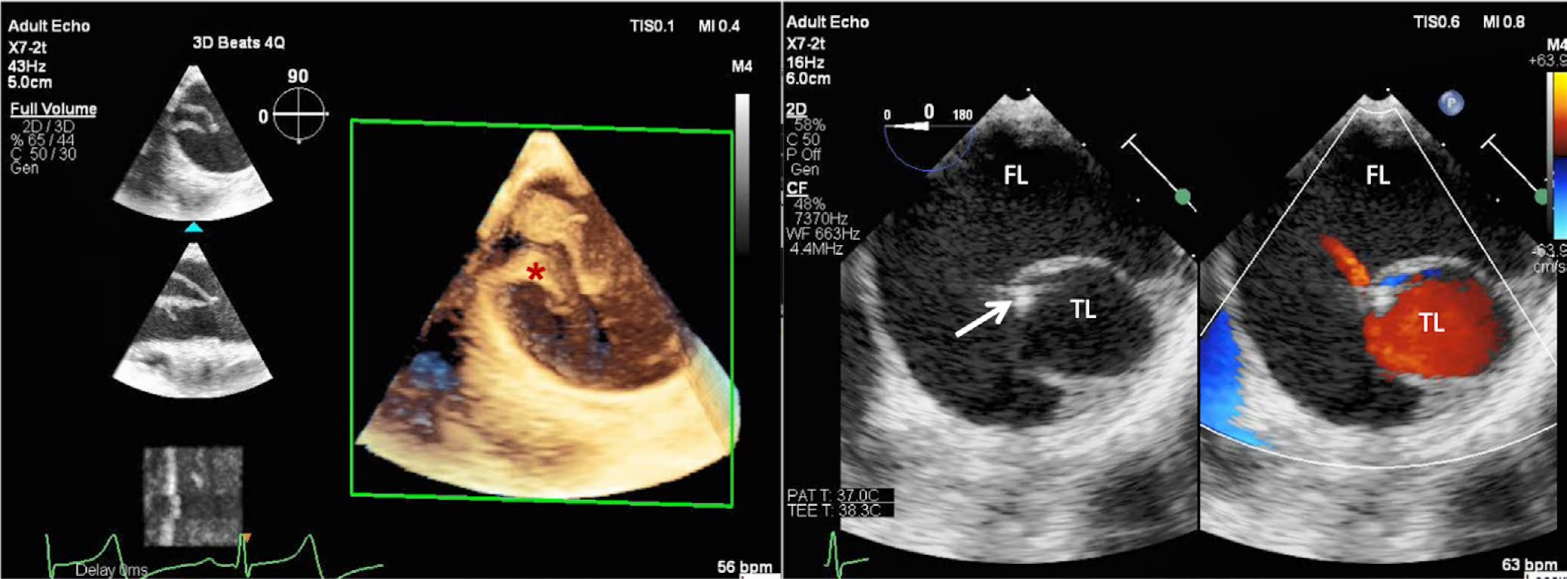
2D and 3D TOE help to identify the true and false lumen and guide the catheter advancing in the true lumen (white arrow). 3D TOE nicely demonstrates the small true lumen and identifies the entry tear (asterisk)

Thoracic Endovascular Aortic Repair was performed under general anesthesia with access obtained from both the left brachial artery by puncture and from the right femoral artery by surgical exposure to enable the insertion of a vascular sheath. An additional venous access was established to place a temporary pacing wire for intermittent rapid pacing at 180 bpm while delivering the stent graft. A bidirectional approach was chosen to navigate the true lumen exclusively (embracing pigtail catheters technique) from proximal (brachial) and distal (femoral) access prior to exchanging for a stiff guiding wire (Figure 2A–D). The “embracing pigtail” navigation in the true lumen was visualized by digital subtraction angiography (DSA) and simultaneous transoesophageal echocardiography (TOE). First, a right coronary artery catheter (JR4, Cordis®) was advanced from the femoral access and navigated into the true lumen under fluoroscopic guidance; selective imaging of the left renal artery, and the two abdominal arteries (superior mesenteric artery and coeliac axis) confirmed position of the catheter in the true lumen (Figure 2C). Second, exchanging the JR4 catheter for a pigtail (Cook Medical Inc.) both pigtails met at the level of diaphragm and embraced each other (Figure 2D). With this “embracing pigtail” maneuver the femoral catheter was safely advanced into the ascending aorta under ultrasound guidance (Figures 2E and 3). Once the femoral pigtail catheter reached the ascending aorta, a Confida™ wire (Medtronic Inc.) was advanced and the pigtail catheter removed.

After this maneuver, a Gore® Dryseal™ 20 French sheath was inserted into the right femoral artery to accommodate a 31 × 200 mm TAG^®^ active control stent graft device (W. L. Gore & Associates, Inc.), which was subsequently advanced and placed at the level of the diaphragm. Using overlay technology from imported CT images and simultaneous ultrasound assessment, the position was optimized, and the stent graft gently deployed under rapid pacing at a systolic blood pressure of about 50 mmHg (Figure 2F). Finally, the delivery system was removed, and final angiography confirmed optimal positioning of stent graft and unblocked left subclavian artery with sealing of all thoracic entry points of the dissection (Figure 2G,H). The patient was transferred to the intensive care and closely monitored overnight. Post‐TEVAR CT imaging demonstrated complete exclusion of the false lumen without malperfusion enabling patient discharge after 5 days (Figure 1C) and scheduled for follow‐up in aortopathy clinic in 6 months.

## 4 DISCUSSION

Thoracic Endovascular Aortic Repair is currently considered the treatment of choice for acute and subacute complicated type B aortic dissection.^4^ However, its role in hereditary connective tissue disorders such as Marfan's syndrome remains controversial due to the higher risk to develop late aortic complications such as retrograde dissection or rupture. Nevertheless, TEVAR has been offered to selected patients with Marfan conditions as a bridge to surgery^5^ or after previous surgical treatment for type A dissection with an interposition graft, which would prevent retrograde dissection.^6^ However, data from large cohorts with reasonable long‐term follow‐up are not available to establish recommendations for patients with known connective tissue disorders. Nevertheless, various articles describe endovascular procedures as a useful bridging concept to elective surgical repair or even instead of open surgery^6^, ^7^, ^8^ in certain conditions.

Our case report is unique for several reasons. First, the disruption of surgical service in 2 major hospitals and limited intensive care capacity in the first wave of the COVID‐19 pandemic impeded the logistics of open surgery for complicated and progressive type B dissection in this case (affording complex redo surgery). Considering the features of a complicated dissection, a swift endovascular management was considered a sensible alternative to surgery; for a safe endovascular procedure, simultaneous use of 3D ultrasound and fluoroscopy was highly instrumental for precise anatomical definition of the true lumen, safe navigation of catheters and quality control of stent graft deployment. In particular, 3D display accurately identifies the entry size dimension and the relation to the spiral dissection (Figure 3).

This case may demonstrate the potential of a complex type B dissection in patients with Marfan's syndrome to be safely managed by improved endovascular technology using low radial force and appropriate imaging support. The clinical scenario with limited access to open surgery and restricted intensive care led to an unconventional but successful endovascular strategy that may be attractive even in post‐pandemic times since our 7‐month follow‐up showed an excellent result and further remodelling without distal expansion of non‐stented abdominal aorta (Figure 1D). Despite the early success, careful surveillance will be warranted by annual clinical follow‐up and imaging in regular intervals. However, more data and experience need to be collected before new recommendations and deviation from standard care can be issued.

## 5 CONCLUSION

This case shows potential for endovascular management of complicated type B aortic dissection in a Marfan patient as feasible alternative to open redo surgery with good short‐term outcome. Both longer follow‐up and more evidence are needed to provide formal recommendations for the use of endovascular approaches in patients with conditions of connective tissue disorders.

## CONFLICTS OF INTEREST

No potential conflicts exist for all authors.

## AUTHOR CONTRIBUTIONS

Conceptualization: UR, CAN. Data curation: XY, MCV, MC. Writing: XY, UR, CAN.

## INFORMED CONSENT

Published with written consent of the patient.

## Data Availability

The data that support the findings of this study are available from the corresponding author upon reasonable request.
